# Developing approaches for linear mixed modeling in landscape genetics through landscape‐directed dispersal simulations

**DOI:** 10.1002/ece3.2825

**Published:** 2017-04-18

**Authors:** Jeffrey R. Row, Steven T. Knick, Sara J. Oyler‐McCance, Stephen C. Lougheed, Bradley C. Fedy

**Affiliations:** ^1^ School of Environment, Resources and SustainabilityUniversity of WaterlooWaterlooONCanada; ^2^ Forest and Rangeland Ecosystem Science CenterU.S. Geological SurveyBoiseIDUSA; ^3^ Fort Collins Science CenterU.S. Geological SurveyFort CollinsCOUSA; ^4^ Department of BiologyQueen's UniversityKingstonONCanada

**Keywords:** fox snake, mixed models, maximum‐likelihood population‐effects models, model selection, Ontario, sage‐grouse, spatial genetic simulations, wyoming

## Abstract

Dispersal can impact population dynamics and geographic variation, and thus, genetic approaches that can establish which landscape factors influence population connectivity have ecological and evolutionary importance. Mixed models that account for the error structure of pairwise datasets are increasingly used to compare models relating genetic differentiation to pairwise measures of landscape resistance. A model selection framework based on information criteria metrics or explained variance may help disentangle the ecological and landscape factors influencing genetic structure, yet there are currently no consensus for the best protocols. Here, we develop landscape‐directed simulations and test a series of replicates that emulate independent empirical datasets of two species with different life history characteristics (greater sage‐grouse; eastern foxsnake). We determined that in our simulated scenarios, AIC and BIC were the best model selection indices and that marginal *R* ^2^ values were biased toward more complex models. The model coefficients for landscape variables generally reflected the underlying dispersal model with confidence intervals that did not overlap with zero across the entire model set. When we controlled for geographic distance, variables not in the underlying dispersal models (i.e., nontrue) typically overlapped zero. Our study helps establish methods for using linear mixed models to identify the features underlying patterns of dispersal across a variety of landscapes.

## 1 Introduction

Identifying the natural and anthropogenic landscape features that promote or impede dispersal provides ecological context for understanding how populations are structured across a landscape (Manel, Schwartz, Luikart, & Taberlet, 2003). Dispersal is critical to local population dynamics (Vance, 1984), and when it results in gene flow (i.e., effective dispersal), it is essential to maintaining genetic diversity (Epps et al., 2005). Thus, a comprehensive understanding of how dispersal is influenced by landscape features can provide insight into the ecological factors underlying patterns of geographic variation and inform management actions designed to improve or sustain the viability of populations. As a result of the importance of dispersal, the last decade has seen a proliferation of quantitative methods that combine landscape modeling with genetic data to test hypotheses regarding the relative influence of landscape factors on spatial genetic structure (Balkenhol, Waits, & Dezzani, 2009; Manel et al., 2003; Storfer et al., 2007). Despite the large number of available methods, the power of many of these approaches has not been adequately tested nor have proper protocols been established.

Pairwise metrics for genetic differentiation and landscape resistance (or cost) are commonly compared to quantify landscape effects on dispersal (e.g., McRae, 2006; Munshi‐South, 2012; Row, Blouin‐Demers, & Lougheed, 2010; Spear, Balkenhol, Fortin, McRae, & Scribner, 2010). Improved model fit between genetic and resistance distance over the fit between genetic and Euclidean distance (i.e., isolation‐by‐distance) suggests a link between the characterized landscape and patterns of effective dispersal. However, the best approach for quantifying model fit and comparing models that represent different hypotheses is far from clear. This lack of clarity largely stems from the nonindependent error structure within pairwise datasets that preclude standard information theoretic model selection approaches. This has led many to use permutation analyses such as Mantel's tests (e.g., Cushman & Landguth, 2010; Cushman, McKelvey, Hayden, & Schwartz, 2006; Schwartz et al., 2009) or multiple regression of distance matrices (MRDM, Manly, 1986). However, Mantel's test are largely limited to a maximum of three matrices (i.e., two independent variables) with a propensity for inflated error rates (Guillot & Rousset, 2013), and nonindependence issues for MRDM make model selection indices prone to bias (Goldberg & Waits, 2010; Van Strien, Keller, & Holderegger, 2012). This limits the use of these approaches for disentangling the ecological complexity surrounding spatial genetic structure.

Maximum‐likelihood population‐effects (MLPE) mixed models include a covariate structure that accounts for the nonindependent error structure of pairwise datasets (Clarke, Rothery, & Raybould, 2002). Briefly, in the model specification each pairwise data point is considered an observation, but the lack of independence is incorporated as a population‐level factor that distinguishes between data points that share a common deme (not independent) and those that do not (independent). These models have recently emerged as a powerful alternative that permits information theoretic model selection (Blair, Jiménez Arcos, Mendez de la Cruz, & Murphy, 2013; Peterman, Connette, Semlitsch, & Eggert, 2014; Phillipsen et al., 2015; Row et al., 2015; Van Strien et al., 2012; Zancolli, Rödel, Steffan‐Dewenter, & Storfer, 2014). A comparison of the fit of models in a biologically relevant model set can readily accommodate ecological complexity (Burnham & Anderson, 2002). However, an evaluation of this approach in the context of landscape genetics is lacking an assessment that is required to determine which model selection indices are most appropriate for distinguishing the importance of landscape variables on dispersal. We used a simulation‐based analysis, parameterized from extensive empirical data, to test the efficacy of model selection indices and to help establish approaches for using MLPE models to identify landscape features that impact dispersal.

We developed landscape‐directed dispersal simulations in which dispersal rates among simulated populations are governed by landscape features from real landscapes and produced genetic characteristics (e.g., genetic diversity and differentiation) similar to empirical datasets. Using these simulations, we derived a series of replicates where genetic exchange among populations for a wide‐ranging terrestrial vertebrate (greater sage‐grouse [*Centrocercus urophasianus*] across Wyoming, ~121,000 km^2^) and a less mobile reptile (eastern foxsnake [*Patherophis gloydi*] across southwestern Ontario ~3,200 km^2^) was simulated and directed by one or more landscape features. With these replicates, we (1) compared the ability of a variety of model selection indices to identify the correct underlying dispersal model (i.e., true dispersal model) from among a candidate set of models and (2) examined the stability of model coefficients for the landscape variables in the true dispersal model (i.e., true landscape variables) across all models in a model set. Our study assists with the development of methods for using MLPE models in a model selection framework to test hypotheses and establish the landscape features that are significantly impacting patterns of dispersal across landscapes. Further, we provide genetic simulation scripts that can be used to test population‐based spatial genetic approaches.

## 2 Methods

### 2.1 Landscape‐directed dispersal simulations

#### 2.1.1 Resistance surfaces for greater sage‐grouse across Wyoming

Maximum‐likelihood population‐effects models require a set of resistance surfaces, where grid cells in a geographic raster coverage represent some measure of movement resistance ranging from neutral to complete impediment. We developed a set of resistance surfaces describing dispersal for sage‐grouse across Wyoming for the simulation model. The first four surfaces were derived from individual landscape components (percent cover of sagebrush (*Artemisia* spp.; SAGE), forest (FOR), agricultural fields (AGRIC), and terrain ruggedness (RUGG); Table 1) important for sage‐grouse functional connectivity across this region (Row et al., 2015). All land cover layers had a resolution of 300 × 300 m cells, and original land cover sources can be found in Row et al. (2015) and Table 1. FOR, AGRIG and RUGG are all inhibitors of gene flow (i.e., higher values equal higher resistance) and thus resistance increased as the percent cover (AGRIC and FOR) or average value (RUGG) increased. SAGE promoted gene flow (i.e., high cover equals low resistance), and thus, raw values were reversed by subtracting each value from the maximum overall value for that surface and adding 0.1 to avoid zero (Row et al., 2014). This gave the lowest resistance to cells with the highest sagebrush cover, and as sagebrush cover decreased, resistance increased. For SAGE, we also set raw values of less than 4% (approximate error for sagebrush cover dataset; Homer, Aldridge, Meyer, & Schell, 2012) to zero, and thus, these were set to the highest resistance value in the transformation to resistance. Resistance surfaces resampled using moving windows to higher spatial resolutions provided a better fit with genetic data (Row et al., 2015). Thus, we replaced individual cell values by the mean within the moving window of 6.44 km (known region of influence for habitat selection and movement; Holloran & Anderson, 2005; Doherty, Naugle, & Walker, 2010; Fedy et al., 2012). Nonzero values were then rank‐transformed to normality using the *GenABLE* package in R (R Core Team 2016). This procedure ranked the resistance values, standardized them to values between 0 and 1, and then used these values as probabilities to transform the ranks to a standard normal distribution (*qnorm* function in R). This standardized both the range and distribution of resistance values, avoided skewed distributions, and facilitated comparisons among variables (Fig. S1). The standardization also was necessary because otherwise pairwise resistances from the combined surfaces (i.e., two averaged resistance surfaces) were not linear combinations of pairwise resistances derived from each of the surfaces individually. For example, given the distribution of values, pairwise resistances derived from unstandardized SAGE and RUGG were highly correlated with SAGE and very different from those derived from RUGG.

**Table 1:** Raw landscape components that were used in the derivation of resistance surfaces for sage‐grouse and eastern foxsnakes

Variable	Raw value description	Source
Sage‐grouse		
FOR	Percent coverage of forest^a^	Northwest ReGAP
SAGE	Percent coverage of sagebrush (all *Artemisia* species combined)^a^	Homer et al. (2012)
AGRIC	Percent coverage of irrigated and nonirrigated agricultural fields^a^	Fedy et al. (2014)
RUGG	Terrain Ruggedness Index: Low values represent flat areas, and high values represent steep and uneven terrain^a^	Sappington, Longshore, and Thompson (2007)
Eastern foxsnake		
OPEN	Percent coverage of open seminatural field and marsh habitat^a^	Row et al. (2010)
WATER	Percent coverage of open water^a^	Row et al. (2010)
ROAD	Density of roads	Row et al. (2010)
RESID	Percent coverage of developed land (urban and residential)	Row et al. (2010)

All raw values were averaged using a 6.44‐km moving window for the sage‐grouse dataset and a 1.5‐km moving window for the foxsnake dataset.

a Promoter of gene flow (i.e., high cover equals low resistance), and thus, raw values were reversed by subtracting each value from the maximum overall value.

**Figure 1:**
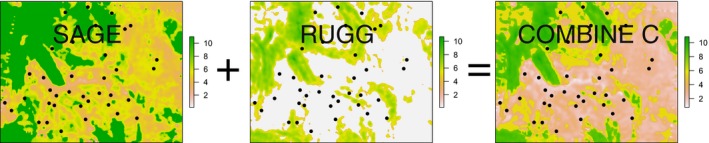
Combined resistances surfaces were developed by averaging the resistance values from two surfaces derived from individual landscape components. Here, we show the derivation of SAGE (percent sagebrush cover) and RUGG (terrain ruggedness). Locations of sage‐grouse lek groupings are shown as black dots, and the overall distribution of cell values can be found in Fig. S1

Dispersal patterns for most species are likely influenced by more than one landscape feature, and thus, we derived six ecologically relevant combined resistance surfaces for Wyoming (A: FOREST and SAGE; B: SAGE and AGRIC; C: SAGE and RUGG; D: FOREST and AGRIC; E: FOREST and RUGG; and F: RUGG and AGRIC). For simplicity, we equally weighted each surface by averaging the two individual component resistance surfaces. Averaging the surfaces (as opposed to adding) merged the features of both components but kept the overall ranges of the resistance values consistent with the single variable surfaces (Figure 1). Pairwise resistance values from combined surfaces that were added together were perfectly correlated with the average surface. Lastly, we used a landscape‐free undifferentiated surface. It should yield similar results as straight‐line distances, but is more appropriate because it has the same landscape boundaries as our other surfaces (i.e., spatial distances assume an unbounded landscape; Lee‐Yaw, Davidson, McRae, & Green, 2009; Row et al., 2010). Thus, we derived 11 resistance surfaces to simulate dispersal among groupings of sage‐grouse in our simulations. Thirty‐seven populations were defined by buffering large leks (~8 km), which are centralized breeding sites where males congregate to compete for females and from which feather samples were collected (Row et al., 2015). For each of the resistance surfaces, we used circuit theory (Hanks & Hooten, 2013; McRae, Dickson, Keitt, & Shah, 2008) as implemented in Circuitscape to calculate pairwise resistance between the 37 lek population groupings (see Figs S1 and S2 of Supporting information for distribution and correlation of resistance surfaces). Pairwise resistance distances were used in both empirical datasets, and this approach accounts for the size, amount, and quality of connecting habitat and thus offers advantages over alternatives that rely on a single low‐cost path (McRae & Beier, 2007). The mean distance between lek groupings was 44 km with a range of 19–78 km, which is within the seasonal movement distances of sage‐grouse (Fedy et al., 2012), making direct dispersal links between neighboring groups likely. Each of the 11 pairwise resistance matrices was used independently as a true landscape dispersal replicate. For all analyses and simulations, all pairwise comparisons were included.

#### 2.1.2 Resistance surfaces for eastern foxsnakes across southwestern Ontario

Our second set of simulations were derived to match an empirical population genetic dataset (324 individuals from 17 populations) for eastern foxsnakes across a fragmented region in southwestern Ontario (Row et al., 2010). Matching Row et al. (2010), we simulated a total of 17 populations distributed across the study site. We summarized the landscape using four individual landscape components derived in Row et al. (2010): percent cover of open seminatural habitat (OPEN), open water (WATER), roads (ROAD), and residential and urban development (RESID; Table 1). All land cover layers had a resolution of 40 × 40 m cells with ROAD and RESID assumed to be inhibitors of gene flow (i.e., higher percent cover equal to higher resistance). OPEN and WATER were assumed to be promoters of gene flow (i.e., high percent cover equals low resistance), and thus, raw values for these surfaces were reversed by subtracting each value from the maximum overall value for that surface and adding 0.1 to avoid zero. We derived resistance surfaces by first transforming raw values using a 1.5‐km moving window (mean of the maximum distance away from hibernation site during an active season; Row, Blouin‐Demers, & Lougheed, 2012), which replaced individual cell values by the mean within the moving window. Again nonzero values were rank‐transformed to normality. Using the raw‐transformed variables resulted in correlated pairwise resistance values. After preliminary simulations, we set very low densities (<4%) to zero for WATER and OPEN and set zero values to a high resistance (value of 20) to derive a set of uncorrelated predictors for the model selection analysis. See Figs S3 and S4 for histograms and correlation plots.

As with the sage‐grouse simulations, we also derived seven, ecologically relevant, combined resistance surfaces (A: OPEN and ROAD; B: OPEN and RESID; C: WATER and ROAD; D: WATER and RESID; E: OPEN and WATER; and F: ROAD and RESID) by taking the average of two individual component resistance surfaces.

#### 2.1.3 Dispersal simulations

We used pairwise resistance matrices to simulate dispersal between population groupings for sage‐grouse and foxsnakes using *simuPOP* (Peng & Amos, 2008; Peng & Kimmel, 2005) modules in Python 2.7.2. For each resistance surface, the pairwise dispersal (Disp_*ij*_; proportion of migrants between populations) between populations was derived from a negative exponential function:

(1) $${\text{Disp}}_{ij}=-\alpha e^{R_{ij}\beta }$$

where Disp_*ij*_ decreases with increasing resistance (*R*_*ij*_), with α determining the steepness of the decline and β describes the overall dispersal rate. We standardized emigration rates (i.e., proportion of individuals migrating from population *i* to population *j*) so that they were consistent among populations. This avoided complete isolation of populations in low‐quality areas, which were resulting in patterns different from our observed data (see below). Biologically, this is equivalent to individuals in poor habitat having a higher threshold and being more willing to travel through high‐resistant habitat (Long, Diefenbach, Resenberry, Wallingford, & Grund, 2005; Martín et al., 2008; Matthysen, Adriaensen, & Dhondt, 1995) (see Fig. S5 for a graphical representation of the transformation). The relationship between dispersal and resistance was consistent for all populations (i.e., populations with lower resistance between them had greater pairwise dispersal).

We simplified the simulations by maintaining the same constant population sizes (N) for all populations across generations within a replicate. We simulated microsatellite loci to match the empirical datasets (14 for sage‐grouse and 11 for foxsnakes); mutation rates (mμ) were assumed to be microsatellites (most current landscape genetic literature is based on surveys of microsatellite markers), mutating under a strict stepwise mutation model. In total, our genetic model, which required a pairwise resistance matrix and four input parameters (Table S1), modulated genetic diversity and differentiation across our simulated populations (Table S2). All simulations were run for 1,000 generations with the *simuPOP* modules set to output genotype data, which were subsequently analyzed in R (R Core Team 2016).

We ensured that our simulations were biologically relevant by parameterizing our landscape‐directed dispersal simulations to generate simulated data with genetic characteristics similar to the empirical datasets. This was accomplished for each of the dispersal replicates by randomly sampling twenty individuals from each simulated population and comparing summary statistics to empirical data (655 individuals from 37 sage‐grouse populations; 324 individuals from 17 foxsnake populations). In total, we used four steps: (1) running preliminary simulations and determining the range of parameter values that produced data approximating the genetic diversity (expected heterozygosity [He], observed heterozygosity [Ho], mean number of alleles [MNA] and differentiation *G*_ST_; Takezaki & Nei, 1996) of the observed genetic data, (2) running an additional 250 simulations with parameter values randomly selected within the range determined in step 1 (Table S1), and (3) retaining the top ten parameter sets that produced genetic summary statistics closest to observed sage‐grouse data (i.e., lowest standardized Euclidean distances; Table S2). We then ran an additional ten replicates for each of the retained parameter sets for 100 replicate simulations (i.e., ten different parameter sets each run 10 times) for each of the 11 landscapes (1,100 replicate simulations).

### 2.2 Assessment of linear mixed models in spatial genetics

Maximum‐likelihood population‐effects models account for nonindependence in a set of *n* pairwise data points by including a random‐effects term for the nonindependent error structure of pairwise datasets (Clarke et al., 2002). In MLPE models, the fixed effects, or explanatory variables, are the pairwise landscape resistance matrices. The random‐effects term accounts for population‐level influence by setting up the covariance structure such that a proportion (ρ_τ_) of the total variance (σ^2^) is due to the correlation between data points that share a common population. Thus, the covariance for *n* that share a common population is ρ_τ_σ^2^ and zero for those that do not (Clarke et al., 2002; Van Strien et al., 2012). The intercept and slopes for the fixed effects and ρ_τ_ are estimated with restricted maximum likelihood (REML, *lme4* package in R), which produces unbiased estimates of model variance for mixed‐effects models (Clarke et al., 2002; Van Strien et al., 2012). Models were also estimated using the *MCMCglmm* package (Hadfield, 2010) with a similar model formulation for the random‐effects term. MCMC models were run for a total of 100,000 MCMC iterations (250,000 burn‐in, 400 thinning) with convergence assessed by comparing coefficients and their intervals (*confint.merMod* command; *lme4* package) across multiple runs.

We assessed the power of MLPE models by quantifying their ability to identify the true dispersal model from alternate models in a complete candidate set for each simulated replicate. For both the sage‐grouse and foxsnake analyses, we considered a candidate model set of 16 models with each of our true resistance surfaces represented in the set (Table 2). Because model selection approaches generally involve a comparison of model fit and an analysis of the significance and stability of model coefficients (Arnold, 2010), we: (1) calculated the average percentile for true dispersal candidate models (i.e., true model within top X percent of models) across replicates and (2) calculated the average coefficient value and associated confidence interval for true (i.e., contained in the true resistance surface) and nontrue landscape variables in all model sets.

**Table 2:** Model sets for models comparing pairwise genetic differentiation (*G*_ST_) to pairwise resistance distance for landscape variables. See Table 1 for description of variables

Model ID	Model
Sage‐grouse models	
1	$G_{{\mathrm{ST}}_{ij}}∼{\text{UNDIF}}_{ij}$
2	$G_{{\mathrm{ST}}_{ij}}∼{\text{FOR}}_{ij}$
3	$G_{{\mathrm{ST}}_{ij}}∼{\text{FOR}}_{ij}+{\text{RUGG}}_{ij}$
4	$G_{{\mathrm{ST}}_{ij}}∼{\text{FOR}}_{ij}+{\text{AGRIC}}_{ij}$
5	$G_{{\mathrm{ST}}_{ij}}∼{\text{FOR}}_{ij}+{\text{RUGG}}_{ij}+{\text{AGRIC}}_{ij}$
6	$G_{{\mathrm{ST}}_{ij}}∼{\text{SAGE}}_{ij}$
7	$G_{{\mathrm{ST}}_{ij}}∼{\text{SAGE}}_{ij}+{\text{RUGG}}_{ij}$
8	$G_{{\mathrm{ST}}_{ij}}∼{\text{SAGE}}_{ij}+{\text{AGRIC}}_{ij}$
9	$G_{{\mathrm{ST}}_{ij}}∼{\text{SAGE}}_{ij}+{\text{RUGG}}_{ij}+{\text{AGRIC}}_{ij}$
10	$G_{{\mathrm{ST}}_{ij}}∼{\text{SAGE}}_{ij}+{\text{FOR}}_{ij}$
11	$G_{{\mathrm{ST}}_{ij}}∼{\text{SAGE}}_{ij}+{\text{FOR}}_{ij}+{\text{RUGG}}_{ij}$
12	$G_{{\mathrm{ST}}_{ij}}∼{\text{SAGE}}_{ij}+{\text{FOR}}_{ij}+{\text{AGRIC}}_{ij}$
13	$G_{{\mathrm{ST}}_{ij}}∼{\text{SAGE}}_{ij}+{\text{FOR}}_{ij}+{\text{RUGG}}_{ij}+{\text{AGRIC}}_{ij}$
14	$G_{{\mathrm{ST}}_{ij}}∼{\text{RUGG}}_{ij}$
15	$G_{{\mathrm{ST}}_{ij}}∼{\text{AGRIC}}_{ij}$
16	$G_{{\mathrm{ST}}_{ij}}∼{\text{RUGG}}_{ij}+{\text{AGRIC}}_{ij}$
Foxsnake models	
1	$G_{{\mathrm{ST}}_{ij}}∼{\text{UNDIF}}_{ij}$
2	$G_{{\mathrm{ST}}_{ij}}∼{\text{OPEN}}_{ij}$
3	$G_{{\mathrm{ST}}_{ij}}∼{\text{OPER}}_{ij}+{\text{ROAD}}_{ij}$
4	$G_{{\mathrm{ST}}_{ij}}∼{\text{OPEN}}_{ij}+{\text{RESID}}_{ij}$
5	$G_{{\mathrm{ST}}_{ij}}∼{\text{OPEN}}_{ij}+{\text{ROAD}}_{ij}+{\text{RESID}}_{ij}$
6	$G_{{\mathrm{ST}}_{ij}}∼{\text{WATER}}_{ij}$
7	$G_{{\mathrm{ST}}_{ij}}∼{\text{WATER}}_{ij}+{\text{ROAD}}_{ij}$
8	$G_{{\mathrm{ST}}_{ij}}∼{\text{WATER}}_{ij}+{\text{RESID}}_{ij}$
9	$G_{{\mathrm{ST}}_{ij}}∼{\text{WATER}}_{ij}+{\text{ROAD}}_{ij}+{\text{RESID}}_{ij}$
10	$G_{{\mathrm{ST}}_{ij}}∼\text{OPEN}+{\text{WATER}}_{ij}$
11	$G_{{\mathrm{ST}}_{ij}}∼\text{OPEN}+{\text{WATER}}_{ij}+{\text{ROAD}}_{ij}$
12	$G_{{\mathrm{ST}}_{ij}}∼\text{OPEN}+{\text{WATER}}_{ij}+{\text{RESID}}_{ij}$
13	$G_{{\mathrm{ST}}_{ij}}∼\text{OPEN}+{\text{WATER}}_{ij}+{\text{ROAD}}_{ij}+{\text{RESID}}_{ij}$
14	$G_{{\mathrm{ST}}_{ij}}∼{\text{ROAD}}_{ij}$
15	$G_{{\mathrm{ST}}_{ij}}∼{\text{RESID}}_{ij}$
16	$G_{{\mathrm{ST}}_{ij}}∼{\text{ROAD}}_{ij}+{\text{RESID}}_{ij}$

If MLPE models have high power to correctly identify true landscape variables, true models should be among the best fitting and thus have a low percentile. Also, because each variable represents pairwise resistance derived from a resistance surface, there should be a positive (i.e., increase in genetic distance with increasing resistance) and significant relationship with genetic distance if the surface is an accurate characterization of the landscape from the perspective of the species. In circuit theory, resistance between populations will increase with increasing distance between nodes regardless of the resistance values of a surface. Thus, in the presence of isolation‐by‐distance, a variable may be spuriously classified as significant even if it does not affect dispersal. To reduce the likelihood of this, we reran all candidate models, but included a variable representing isolation‐by‐distance (resistance calculated using an undifferentiated landscape, UNDIF). Inclusion of this variable should factor out the effects of distance and reduce the percentage of nontrue landscape variables being significant.

The best model selection criteria for MLPE models are unknown; thus, we compared the results of five different criteria designed to estimate the overall model fit of a set of candidate models. First, we used information theoretic criteria, AIC and BIC, which were estimated from the *lme4* models, and DIC values were calculated from the MCMC iterations. We used three different AIC variants, but all gave similar results, so only AIC_REML_ was reported. Next, we used two marginal *R* ^2^ variants, which measured the total variance explained by the fixed effects (i.e., pairwise resistance matrices). Unlike traditional *R* ^2^ values, marginal *R* ^2^ do not necessarily increase with the addition of parameters (Orelien & Edwards, 2008); thus, it has been suggested that marginal *R* ^2^ is suitable for use as model selection criteria (Van Strien et al., 2012). The first *R* ^2^ variant considered was $R_{\beta }^{2}$, which uses the *F* distribution (estimated using Kenward‐Rogers approximation: *KRmodcomp* R package; Halekoh & Højsgaard, 2011) to quantify the difference in explained variation between models with and without fixed effects (Edwards & Muller, 2008). The second, $R_{\mathrm{GLMM}(m)}^{2}$, estimates the variance of the fixed effects by calculating the variance of fitted values predicted from a model with only fixed effects (Nakagawa & Schielzeth, 2013).

We further assessed the top model selection index by calculating (1) the overall proportion of simulations where the top model was the true underlying dispersal model, (2) the proportion of simulations where all true variables were in the top selected model, (3) average difference between the top selected and true model (i.e., delta values for true model), and (4) average correlation between the top selected and true model when the true model was not selected. These comparisons were conducted using all true underlying dispersal scenarios and averaged across replicates.

## 3 Results

### 3.1 Assessment of linear mixed models in spatial genetics

**Figure 2:**
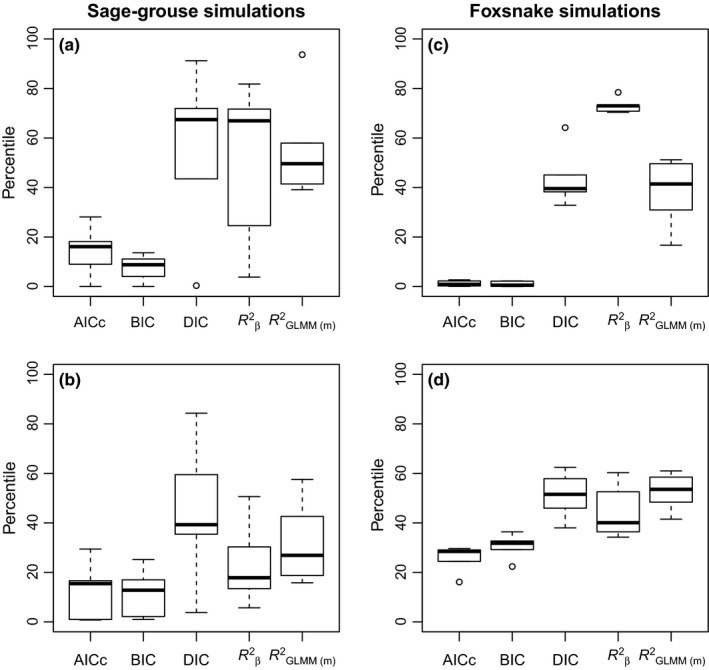
Range of model selection criteria percentiles for (a, c) single and multiple (b, c) variable true models with lower values indicating the true model was found in a higher percentile for a given model selection criteria (i.e., lower values = greater predictive power). Results for simulations emulating the sage‐grouse and foxsnake datasets are shown

Across all replicates, the percentiles for true dispersal models varied depending on the model selection criteria used to compare model fit. In our assessment, lower percentiles represented better performance of the selection criteria (i.e., greater accuracy). The model selection indices AIC and BIC outperformed the other indices and had true models within the lowest percentiles for the total model set in both simulated datasets (Figure 2). Marginal *R* ^2^ values had a bias toward multivariate models and had much higher percentiles for true models than the AIC and BIC model selection criteria. DIC also performed poorly and generally had higher percentiles than marginal *R* ^2^ values (Figure 2). Considering BIC, which performed the best, the true landscape model was always within the top 20% of the 16 models in the set when there was only one underlying landscape variable (Figure 2a,c). When the underlying true dispersal model contained two landscape components, all criteria had higher percentiles for the true models (Figure 2b). However, for AIC and BIC, the true model was still within the top 20% of models for the majority (~75%) of the sage‐grouse simulations. In the foxsnake simulations, the AIC and BIC similarly had had a superior performance for both univariate and multivariate models (Figure 2b,d). However, there was a greater drop off in performance for the multivariate landscape models, with the true models only within the top 30% of models.

The performance of AIC and BIC was very similar, and thus, here we only discuss BIC results which performed slightly better. The proportion of correctly assigned true models varied widely between dispersal scenarios and was not high overall (Table 3). In general, the correct assignment proportions and ΔBIC values were much lower for single landscape models. When the correct model was not identified, the correlation in pairwise resistances derived in the top and true model was high with an average of 0.77 and 0.71 for the sage‐grouse and foxsnake datasets, respectively.

**Table 3:** Performance of model selection analysis with BIC as an indicator showing variable results depending on the underlying simulations

Model	Proportion of correct model selection	Proportion tests with all true variables in top model	Mean ΔBIC	Mean correlation of top and true model
Sage‐grouse simulations				
UNDIF	1.00	1.00	0.00	NA
AGRIC	0.34	1.00	−4.33	0.78
FOR	0.37	0.40	−7.56	0.42
RUGG	0.08	1.00	−9.15	0.83
SAGE	0.00	1.00	−15.00	0.82
COMBA	0.86	0.92	−0.33	0.88
COMBB	0.00	0.06	−16.49	0.78
COMBC	0.05	0.05	−6.67	0.84
COMBD	0.89	0.89	−0.25	0.78
COMBE	0.34	0.34	−4.87	0.66
COMBF	0.07	0.97	−12.71	0.92
Foxsnake simulations				
UNDIFF	0.70	0.70	−0.63	0.36
OPEN	1.00	1.00	0.00	NA
ROAD	0.99	1.00	0.00	0.75
RESID	0.64	0.64	−1.48	0.61
WATER	0.91	0.92	−0.25	0.68
COMBA	0.00	0.00	−12.68	0.81
COMBB	0.00	0.00	−7.58	0.83
COMBC	0.00	0.00	−18.82	0.55
COMBD	0.00	0.00	−10.05	0.80
COMBE	0.00	0.00	−8.84	0.87
COMBF	0.00	0.00	−10.43	0.91

The proportion of tests where the top model was the true model, the proportion of simulation where all true variables were in the top model, and average ΔBIC values and the correlation in pairwise resistance values between the top and true model are shown. Average correlation was only calculated for tests where the top model and true model were not the same.

**Figure 3:**
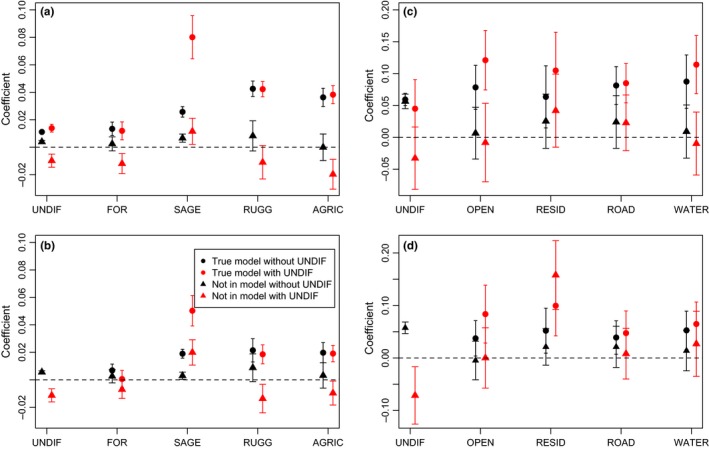
Average model coefficients for variables in and not in the true dispersal simulation with UNDIF_*ij*_ included and not included in all models (UNDIF only model always had UNDIF included). Mean coefficients and upper and lower confidence intervals are shown, and *SD*s of replicates for single (a, c) variable true models and multivariable (b, d) models. Results for simulations emulating the sage‐grouse (a, b) and foxsnake (c, d) datasets are shown

When considering single landscape true models, the average coefficient estimates across models for the true landscape variables (including all replicates and models without UNDIF) were positive and nonoverlapping with zero (Figure 3a,c). As predicted, when UNDIF was included as a variable in the model, true landscape variables remained the same, whereas nontrue landscape variables were reduced and generally fell below zero (Figure 3a,c). For the multilandscape variable dispersal models, the coefficients for true variables were lower, but the patterns were generally consistent (Figure 3b,d). There were, however, exceptions in both the sage‐grouse and foxsnake simulations. In the sage‐grouse multilandscape simulations, including UNDIF reduced the FOR variable coefficient so that it overlapped zero even when it was a true variable in the dispersal model (Figure 3b). In the foxsnake multilandscape simulations, including UNDIF in the model did not reduce the RESID coefficient as it did with the other nontrue landscape variables (Figure 3d).

For both the sage‐grouse and foxsnake simulations, the coefficient for UNDIF was similar and positive whether it was the true landscape variable or not. However, in both cases the average across models decreased below zero when included in a model with true landscape variables (Figure 3) and stayed positive when the true landscape variable was the UNDIF landscape.

## 4 Discussion

Model selection criteria for MLPE models varied in their capacity to identify the true landscape dispersal surface among a candidate set of models. Both AIC and BIC outperformed the other tested indices, with marginal *R* ^2^ values being biased toward more complex models in our simulations. However, even these model selection indices had difficulty in selecting the true dispersal surface when the dispersal was the results of a combination of multiple landscape surfaces. Model coefficients for true landscape variables generally had confidence intervals that did not overlap with zero for the entire model set. Controlling for geographic distance by including an undifferentiated covariate increased model accuracy by reducing the significance of variables not included in the true landscape surface driving dispersal. Overall, a MLPE approach using univariate models that combined AIC and BIC selection indices with an examination of coefficient stability gave the highest likelihood of correctly identifying the true landscape surfaces underlying dispersal.

### 4.1 Model selection indices

Some have suggested model selection indices such as AIC and BIC are inappropriate for mixed models with different fixed effects that are estimated with REML (Verbeke & Molenberghs, 2000). This could be problematic, as REML is used for MLPE models (Clarke et al., 2002), and thus, Van Strien et al. (2012) suggested instead using $R_{\beta }^{2}$ to select the top models. This selection index has subsequently been used by others for landscape genetics (Blair et al., 2013; Phillipsen et al., 2015). However, our results indicate that marginal *R* ^2^ values are biased toward more complex models and often resulted in the selection of multiparameter models, even when a single landscape component was responsible for the underlying dispersal patterns. This is perhaps not surprising, as marginal *R* ^2^ values do not actually decrease with overfitting, but fail to increase with the addition of uninformative covariates (Orelien & Edwards, 2008). This does not lend itself to being an ideal model selection index, and combined with our results, we suggest the use of *R* ^2^ values alone should not be used to select among dispersal models in isolation‐by‐resistance analysis.

Despite the potential issues, AIC can be informative as a model selection index for mixed‐model fit with REML (Gurka, 2006), and they have been used successfully for model selection with MLPE models (Emel & Storfer, 2014; Peterman et al., 2014; Zancolli et al., 2014). Indeed, we found that both AIC and BIC were superior to the other tested selection indices. In the univariate analyses, the true dispersal models were within the top 20% of the model set. However, the percentages of correctly identifying the top model and ΔAIC and ΔBIC values varied dramatically among the true model surfaces and was low overall. When model selection was derived from a single landscape variable, the true landscape variable was generally within the top model and thus, combining model selection using BIC or AIC with coefficient analysis would likely lead to the lowest error rates for our simulations.

When the underlying true landscape resistance was built from two surfaces, the model selection indices were not as proficient at selecting the correct multivariate model. This is likely due to the fact we assumed that pairwise resistances derived from the combination landscape surfaces are a linear combination of the pairwise resistance from each individual landscape. We tested this by fitting models of pairwise resistances from combined surfaces with their corresponding individual pairwise resistances (e.g., Combine A ~ SAGE + FOREST for the sage‐grouse resistance). If the surfaces are linear combinations, we would predict a strong fitting model with both variables contributing relatively equally to the model. For the combined sage‐grouse resistances, this was generally true with an average *R* ^2^ of 0.98 and very similar standardized model coefficients. However, the foxsnake resistances, where multivariate models had a greater difficulty with combined surfaces, the fit was not as high (average *R* ^2^ = .83), and in some cases, one variable's contribution to the model outweighed the other by as much as a factor of four. This is also supported by the fact that in the combined surface simulations, the top models were highly correlated with the true models when not correctly chosen.

In our development of resistance surfaces, we made efforts to develop uncorrelated resistance surfaces that could be combined without masking the effects of one of the landscape variables. This might be difficult with some empirical datasets, particularly when equal contributions of each landscape are not expected as we simulated here. Based on our results, we suggest testing resistances derived from multilandscape surfaces against the corresponding single landscape surfaces before proceeding with multivariate models. More simply, using resistances derived from the combined surfaces in the model selection analyses and avoiding multivariate models altogether might lead to better results.

### 4.2 Coefficient analyses of MLPE models

Many examples of MLPE models in landscape genetics present only model selection indices and do not report model coefficients (Blair et al., 2013; Peterman et al., 2014; Phillipsen et al., 2015). Our results suggest that an analysis of the confidence intervals for landscape variables can support and add to the model selection results. We found that in most cases, the coefficients for true landscape variables were positive and did not overlap zero for all models in which they appeared. They were also generally higher than when they were not in the underlying dispersal model. However, nontrue variables were also significantly positive in many cases. Examination of models that control for distance could help address the selection of false‐positive variables. When we included pairwise resistance values from an undifferentiated landscape, the confidence intervals of nontrue variables typically overlapped with zero or became negative. Thus, confirming the stability of coefficients for landscape components in top models when incorporated into a model that controls for distance will reduce the likelihood of falsely identifying variables as influencing gene flow.

### 4.3 Future considerations to test and refine MLPE approaches

In this study, we used a comparative approach and found relatively consistent results across two datasets designed to emulate species with vastly different life history characteristics. This implies some generality to our results and that using AIC or BIC and examining model coefficient stability will improve the efficiency of MLPE models and reduce error rates. However, we made simplifying assumptions, and more research is required to refine the approaches suggested here and to estimate and compare potential error rates with other landscape genetic approaches. In our simulations, we assumed fixed population sizes, nonoverlapping generations, complete population sampling, and constant dispersal on the landscape. This is oversimplified from empirical data, and adding variation into any of these parameters would obviously introduce noise and make it more challenging to identify the true underlying patters. Thus, to quantify meaningful error rates, more complex simulations including the addition of noise in each of these parameters are needed. Further, comparing the results using different sampling strategies (e.g., population vs. individual), types and the numbers of genetic markers, and other approaches such as Mantel tests and multiple regression on distance matrices (MRDM) would be illuminating.

The importance of simulation software that can simulate dispersal driven by landscape resistance is seen with CDPOP (Landguth & Cushman, 2010), which has been instrumental in testing landscape and population genetic assumptions (e.g., Cushman & Landguth, 2010; Dileo, Rouse, Dávila, & Lougheed, 2013; Landguth et al., 2012; Oyler‐McCance, Fedy, & Landguth, 2012) or predicting the impacts of environmental changes (Row et al., 2014). Here, we have used population‐based simulations to test model selection indices and coefficient analyses. We provided Python and R scripts and a simplified tutorial (see Appendix S1) for using our dispersal model to simulate genetic exchange between populations based on pairwise resistance and to analyze the output using MLPE modeling. We hope that these resources will facilitate the use of simulations to test empirical model selection analyses or other population‐based approaches in spatial genetics.

## Conflict of Interest

None declared.
