# Supplementary material for: Developing approaches for linear mixed modeling in landscape genetics through landscape‐directed dispersal simulations
Source: Ecol Evol. 2017 Apr 18;7(11):3751–61. doi: 10.1002/ece3.2825 (PMC5468135; doi:10.1002/ece3.2825)
Supplement: Supplementary file 1 [file ECE3-7-3751-s001.docx]

**Figure S1.** Distributions of resistance values for individual landscape component raster used in sage-grouse simulations. Because values of SAGE were reverse, zero values are set to the maximum resistance.

**Figure S2.** Correlation between pairwise resistance values derived from landscape component resistance surfaces and an undifferentiated landscape.

**Figure S3.** Distributions of resistance values for individual landscape component raster used in foxsnake simulations.

**Figure S4.** Correlation between foxsnake pairwise resistance values derived from landscape component resistance surfaces and an undifferentiated landscape.

**Figure S5.** Example of relationship between pairwise resistance and emigration (with and without standardizing for the overall rates between populations) for a well connected (a) and isolated (b) population. Without standardization the unconnected population would be highly differentiated from all populations given the low dispersal rates.
