# Supplementary material for: Developing approaches for linear mixed modeling in landscape genetics through landscape‐directed dispersal simulations
Source: Ecol Evol. 2017 Apr 18;7(11):3751–61. doi: 10.1002/ece3.2825 (PMC5468135; doi:10.1002/ece3.2825)
Supplement: Supplementary file 2 [file ECE3-7-3751-s002.docx]

**Table S1.** Model parameters used in simplified genetic model, which simulates genetic exchange between equally size populations according to pairwise resistance. Prior distribution of ranges in used in initial sage-grouse (top) and foxnsake (bottom) simulations

| **Parameter** | **Description** | **Lower** | **Upper** |
| --- | --- | --- | --- |
| N | Population sizes. Was the same for all populations and held constant through time | 800  100 | 1000  600 |
| *mμ* | Mutation rate of genetic loci. Was the same for all loci. | 0.005  0.005 | 0.008  0.008 |
| **α** | Steepness in the decline of dispersal rates as resistance increases (Fig 1; eq 1). | 0.01  0.001 | 0.2  0.4 |
| **β** | Scale of overall dispersal rates (Fig 1; eq 1) | 6  2 | 8  8 |

**Table S2.** Summary statistics calculated for observed and simulated data. The parameters for simulations with the lowest Euclidean distance to observed data were utilized to develop replicated simulations used to test linear mixed modeling in landscape genetics.

| **Summary Statistic** | **Description** |
| --- | --- |
| meanDif | Mean value of genetic differentiation metric. |
| medianDiff | Median value of genetic differentiation metric. |
| maxDiff | Max value of genetic differentiation metric. |
| minDiff | Min value of genetic differentiation metric. |
| lmCoef | Coefficient of linear mixed model describing genetic differentiation with resistance |
| lmSE | Standard deviation of coefficient of linear mixed model describing genetic differentiation with resistance. |
| lmTvalue | T-value of coefficient of linear mixed model describing genetic differentiation with resistance. |
