# Supplementary material for: Developing approaches for linear mixed modeling in landscape genetics through landscape‐directed dispersal simulations
Source: Ecol Evol. 2017 Apr 18;7(11):3751–61. doi: 10.1002/ece3.2825 (PMC5468135; doi:10.1002/ece3.2825)
Supplement: Supplementary file 3 [file ECE3-7-3751-s003.docx]

**Appendix S1:** Using *simuPop* and *R* scripts to run population based simulations and run a model selection analysis with MLPE models.

In this appendix we describe the steps required to run genetic simulations and to identify the landscape variables influencing gene flow using maximum-likelihood population-effects (MLPE) models with a defined model set. Along with this appendix, scripts and example files for running an example analysis (Appendix_S1_sims.zip) are available from the corresponding author.

**Installation of required software:**

In order to run the simulation and MLPE scripts you will need to install R (http://www.r-project.org), Python (https://www.python.org) and the python model simuPOP (http://simupop.sourceforge.net) with its required dependencies. To run R you will also need all packages listed at the beginning of each R script file.

**Setup input files that need to be created or developed:**

There are a number of input files that need to be filled out or developed prior to running a simulation analysis. Below is a list and description of required input files. Within each of these files you will find details on each of the individual parameters that will need to be changed before conducting an analysis.

1. *params.py*: input parameters for running simulations. The parameters need to be set prior to running an analysis. It is likely the prior distributions will need to be modified and tested to ensure they give meaningful results.
2. *Pairwise resistance matrices*: These are contained in a folder *Resistances* with the naming convention as ‘variablename’ and ‘resistances’ separated by an underscore. Included within this list should be resistance values derived from an undifferentiated landscape and must be named (‘*undiff_resistances’*)
3. *Models.r*: a model list containing the model set to test. One of the models must be the true resistance surface and identified in the *params.py* input file. The variable names must match with the resistance files.

**Script files used in the simulations:**

Python scripts

1. *SpatialMigReps_full.py:* Full Python script for running simulations.

R scripts

1. *Rscripts:* R functions required for the analysis.
2. *Model_Selection:* R code for calculating mixed model stats from simulations. The script outputs 3 files (*coefsumfull.csv, coefsumfull_ud.csv, modelsumfull.csv*), which are described below.

**Running a simulation analysis**

To run the complete scripts, first open a terminal window and navigate to the folder containing your scripts and data. Start python (Mac and Linux: type *python* in the terminal window). Then import the main script file:

*import SpatialMigReps_full_160722 as SpMig*

This will load the python module (named as *SpMig* in the workspace) and give you access to the function (*simuSpatial*) to run the analysis. If simuPOP is not installed properly you will receive an error. If you receive no error messages then you can run the simulations and analysis using the following command:

*SpMig.simuSpatial(nreps=5,stand=’NA’)*

This runs the simulations and outputs genepop files with the resulting simulations in the output folder. This will run the simulations without standardized migration rates. Using *stand=’TRUE’* will standardize the migration rates (see manuscript).

**Model selection analysis on simulated data**

Using the script Model_Selection.R you can run a model selection analysis on the simulations or empirical data. For the simulations you can just change your working directory to the Appendix_S2_sims folder and source the script file:

*source(‘Model_Selection.R’).* This script file, 1) imports the model list information from models.R, imports the genetic data and runs and summarizes a model selection analysis and output three files:

1. *coefsumfull.csv:* coefficient summary for linear mixed models summarizing the coefficient estimates and their confidence intervals for each model.
2. *coefsumfull_ud.csv:* coefficient summary for linear mixed models where all models include pairwise resistance values from an undifferentiated landscape.
3. *modelsumfull.csv:* summary of over all fit of each model using BIC.

These summary data files summarize the information for each of the simulated genepop data files in the folder.

**Model selection analysis on empirical data**

You can also run the analysis with empirical data by replacing the simulated data in the output folder with empirical data and running the model selection script. The empirical data must be in genepop format. Further, the pairwise resistance data in the Resistances folder must be replaced and the number of populations must match with the number of populations in the genepop file. Lastly, the models script must be change to the models that are to be compared and the variable name must match the resistance data. For example, if the variable name is ‘open’ in the models script, the corresponding pairwise resistance file in the Resistance folder should be ‘open_resistance’.
